# Deep scoping: a breeding strategy to preserve, reintroduce and exploit genetic variation

**DOI:** 10.1007/s00122-021-03932-w

**Published:** 2021-08-13

**Authors:** David Vanavermaete, Jan Fostier, Steven Maenhout, Bernard De Baets

**Affiliations:** 1 KERMIT, Department of Data Analysis and Mathematical Modelling, Ghent University, B-9000 Ghent, Belgium; 2 IDLab, Department of Information Technology, Ghent University - imec, B-9052 Ghent, Belgium; 3 Progeno, B-9052 Ghent, Belgium

## Abstract

**Key message:**

The deep scoping method incorporates the use of a gene bank together with different population layers to reintroduce genetic variation into the breeding population, thus maximizing the long-term genetic gain without reducing the short-term genetic gain or increasing the total financial cost.

**Abstract:**

Genomic prediction is often combined with truncation selection to identify superior parental individuals that can pass on favorable quantitative trait locus (QTL) alleles to their offspring. However, truncation selection reduces genetic variation within the breeding population, causing a premature convergence to a sub-optimal genetic value. In order to also increase genetic gain in the long term, different methods have been proposed that better preserve genetic variation. However, when the genetic variation of the breeding population has already been reduced as a result of prior intensive selection, even those methods will not be able to avert such premature convergence. Pre-breeding provides a solution for this problem by reintroducing genetic variation into the breeding population. Unfortunately, as pre-breeding often relies on a separate breeding population to increase the genetic value of wild specimens before introducing them in the elite population, it comes with an increased financial cost. In this paper, on the basis of a simulation study, we propose a new method that reintroduces genetic variation in the breeding population on a continuous basis without the need for a separate pre-breeding program or a larger population size. This way, we are able to introduce favorable QTL alleles into an elite population and maximize the genetic gain in the short as well as in the long term without increasing the financial cost.

**Supplementary Information:**

The online version contains supplementary material available at 10.1007/s00122-021-03932-w.

## Introduction

Truncation selection is often used in genomic selection to rapidly increase the short-term genetic gain of a breeding population. By selecting individuals with the highest genomic estimated breeding values (GEBVs), breeders hope to maximally pass favorable properties to their offspring. The underlying idea is easy to understand and matches the gut feeling of most breeders, making it one of the most popular strategies in plant breeding. Unfortunately, truncation selection is also associated with a loss in genetic variation (Jannink 2010). Besides entailing the loss of favorable QTL alleles from the breeding population, truncation selection causes a premature convergence of the genetic value, reducing the long-term genetic gain (Vanavermaete et al. 2020). Therefore, truncation selection can only promise a temporary, short-term increase in the genetic gain. To ensure a continuous increase in the genetic value, new selection methods are needed that maximize both the short-term and the long-term genetic gains.

Different variants of truncation selection that try to remedy the loss in genetic variation have already been proposed in the literature. One way to achieve this is by weighting the marker effects of favorable or low-frequency marker alleles and thus reducing the risk of eliminating important QTL alleles during breeding (Jannink 2010; Liu et al. 2015). The genetic variation can also be preserved by avoiding the selection of closely related individuals as in the *population merit* method (Lindgren and Mullin 1997) or by penalizing the GEBV when two parents with high coancestry are selected as in the *maximum variance total* method (Cervantes et al. 2016). The latter was further improved upon by also minimizing the rate of inbreeding, thus controlling the allele heterozygosity as well as the allele diversity (Brisbane and Gibson 1995; Akdemir and Sánchez 2016). In another strategy, the GEBV was replaced by the *criterion of usefulness* (UC), which not only takes into account the mean predicted genetic value of the offspring, but also the selection intensity, prediction accuracy and genetic variation of the offspring (Lehermeier et al. 2017). The *scoping* method combines pre-selection with a score function to avoid the selection of individuals with a too low GEBV while preserving genetic variation of the breeding population, thus maximizing the long-term genetic gain (Vanavermaete et al. 2020). Whereas the GEBV is based on the total sum of the additive marker effects, the *optimal haploid value* (OHV) scores individuals based on their haplotypes, and can therefore better preserve favorable QTL alleles in the breeding population, increasing the long-term genetic gain (Daetwyler et al. 2015). Müller et al. (2018) propose the expected maximum haploid breeding value (EMBV) to evaluate the potential of a candidate by measuring a limited number of gametes of each parent. The optimal cross selection (OCS) scores a crossing block based on the mean predicted genetic value of the offspring, but also constrains the loss in genetic diversity of the offspring (Akdemir and Sánchez 2016; Gorjanc et al. 2018).

**Fig. 1:**
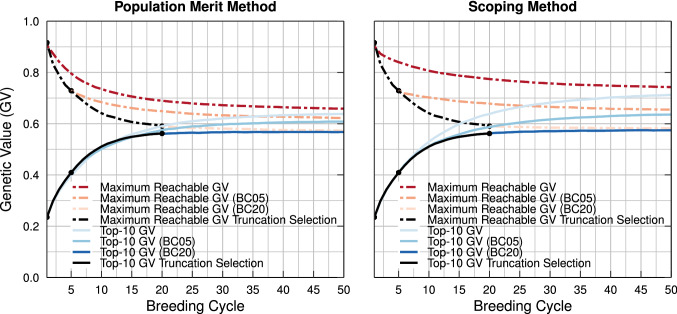
Mean genetic value (GV) of the top-10 individuals in the breeding population using the population merit method (left) and the scoping method (right) after first applying 0, 5, or 20 breeding cycles (BC) of truncation selection (black line). When the genetic variation of the breeding population is already reduced by means of truncation selection, both the population merit method and the scoping method result in a lower genetic value

Unfortunately, the aforementioned methods are generally tested on breeding populations that demonstrate a broad genetic variation. In reality, however, the genetic variation present in most breeding populations has been eroded to some extent by years of consecutive truncation selection. In such cases, the options to further increase the genetic value in the breeding population are strongly reduced. To demonstrate this, we simulate three breeding populations that suffer, to a varying degree, from reduced genetic variation by applying respectively 0, 5, and 20 breeding cycles of truncation selection. Next, using these three breeding populations as a starting point, the performances of the population merit method (Lindgren and Mullin 1997) and the scoping method (Vanavermaete et al. 2020) are compared. When these methods are initiated at a later point, the maximum reachable genetic value of the breeding population is lower, indicating that during truncation selection, favorable QTL alleles have been eliminated from the breeding population (see Fig. 1). Both methods will only be able to preserve a fraction of the genetic variation that is still present in the breeding population. Therefore, the added value of these methods is dramatically reduced when the genetic variation in the breeding population is limited.

When the genetic variation has already been substantially reduced, a gene bank could be used to (re)introduce alleles and haplotypes into the breeding population, resulting in an increase in the maximum reachable genetic value. A gene bank is an (inter)national collection of different plants ranging from wild specimens to different crop varieties at different stages of selection. To optimally reintroduce genetic variation into a breeding population and thus increase the genetic gain in the long term, the gene bank must show a broad genetic variation (Simmonds 1993; Salhuana and Pollak 2006). The introduction of gene bank accessions into the breeding population generally implies a reduction in short-term genetic gain. Depending on the available germplasm collection of the gene bank, different methods have been proposed to introduce such individuals into an elite breeding population. When a phenotypic trait is controlled by only a few genes with large effects, the favorable genes can be introgressed in the breeding population using marker-assisted backcrossing (Han et al. 2017; Smith and Beavis 1996). However, this proved unsuccessful when the phenotypic trait is controlled by many genes of small effect, which is the case for quantitative traits such as grain yield (Bouchez et al. 2002). In this setting, genomic selection (GS) can be used to rapidly introduce (new) QTL alleles from a gene bank into the breeding population (Bernardo 2009). Different mating designs use multi-parental crosses to combine elite individuals with donor individuals selected from a gene bank (Allier et al. 2019; Schopp et al. 2017). Gene bank accessions are first intercrossed to increase the frequency of favorable alleles before they are introduced into the breeding population. Cramer and Kannenberg (1992) proposed a five-year open-ended hierarchical breeding program (HOPE) to introduce new wild specimens into the breeding population using three consecutive gene pools. The HOPE method allows to effectively pass on favorable QTL alleles from the gene bank to the elite breeding population, but the need for additional pre-breeding populations drives up the total cost of the breeding program.

Allier et al. (2020a) recently proposed a new selection method, combining the *haploid estimated breeding value* (HEBV) and the UC to select and cross elite individuals with donor individuals. However, the calculation of the UC requires the construction of a covariance matrix, which considerably increases the computational requirements of the simulation while the bridging population can only reintroduce a fraction of the genetic variation into the breeding population. The parental selection can also be guided using *genotyping-by-sequencing* or related techniques, in which the relatedness of germplasm collections and elite individuals in the breeding population can be quantified and used to preserve the genetic variation in the breeding population (Glaubitz et al. 2014; Gouesnard et al. 2017). The genetic variation of a breeding population can also be increased by using exotic material, but a higher investment is needed to successfully incorporate those alleles in an elite breeding population (Salhuana and Pollak 2006; Wu et al. 2016).

We propose a new method that incorporates the use of a gene bank to reintroduce genetic variation into the breeding population, maximizing the long-term genetic gain without reducing the short-term genetic gain. By using a fraction of the breeding population for pre-breeding, the sizes of both the breeding population and the parental population remain unchanged, avoiding additional costs. This method, coined *deep scoping*, divides the breeding population into an elite population and different layers of pre-breeding individuals. The elite population contains the accessions that have the highest GEBVs and delivers high short-term genetic gain. In the first layer (Layer 0), individuals from a gene bank are crossed with individuals of the elite population, reintroducing genetic variation in the breeding population. Next, different layers are added, allowing for a gradual flow of favorable QTL alleles from the first layer to the elite population. Over each layer, the genetic variation is exploited, increasing the genetic gain and maximizing the transition of pre-bred individuals into the elite population.

## Materials and methods

We adopt the base population and breeding scheme of Neyhart et al. (2017). The base population consists of two datasets of North American barley (*Hordeum vulgare*) from the University of Minnesota (UMN) and the University of North Dakota (NDSU), counting, respectively, 384 and 380 six-row spring inbred lines with 1590 biallelic SNP loci. The same base population was also used by Vanavermaete et al. (2020), ensuring that the performance of the deep scoping method can be compared with that of the scoping method. The parental selection methods are compared using four base populations that differ in their available genetic variation for a single trait of interest. These four base populations (referred to as Population BC05, Population BC10, Population BC15 and Population BC20) are created by reducing the genetic variation using truncation selection in a recurrent breeding scheme for, respectively, 5, 10, 15, and 20 breeding cycles.

**Fig. 2:**
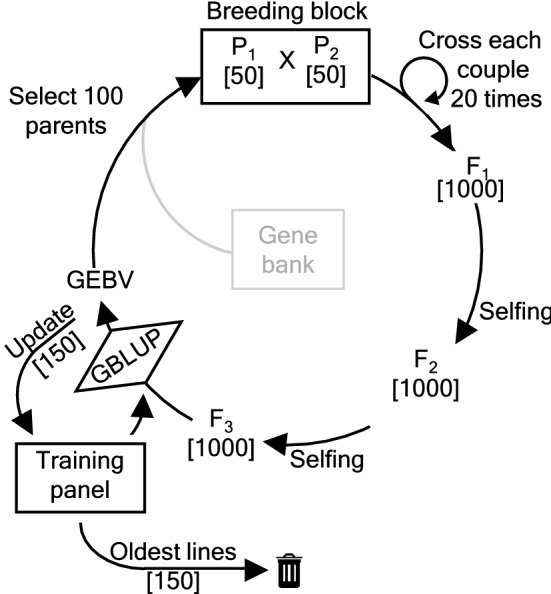
Overview of the recurrent breeding scheme. First, 50 couples of parents ($P_1$, $P_2$) each produce 20 offspring yielding a total of 1000 F1-hybrids. Then, after two generations of single-seed descent, 1000 F3-individuals are obtained. From those F3-individuals, new parental lines are selected. Three different parental selection methods are considered: (i) Truncation selection selects 100 parents with the highest GEBVs and crosses them randomly; (ii) The deep scoping method introduces new genetic information into the breeding population while maximizing the short- and long-term genetic gain; (iii) The HUC method with bridging introduces new genetic information into the breeding population by means of a bridging population

### Breeding scheme

The recurrent breeding scheme shown in Fig. 2 has been described by Vanavermaete et al. (2020) as well as by Neyhart et al. (2017). In this paper, minor modifications are made to this scheme. Over the first breeding cycles, the recurrent breeding scheme is used to decrease the genetic variation of the breeding population. Starting at breeding cycle 0, based on phenotypic data, the top-50 individuals of the NDSU dataset are crossed with the top-50 individuals of the UMN dataset. In the subsequent breeding cycles, the parental selection is completely based on GEBVs, reducing the financial cost of phenotyping. The GEBVs are predicted based on a linear mixed effects model (see Sect. Prediction model). In the recurrent breeding scheme, each parental couple is crossed 20 times, creating in total 1000 F1-hybrids. The F3-individuals are obtained after two cycles of single-seed descent. The recurrent breeding scheme is used to reduce the genetic variation of the breeding population by using truncation selection over 5, 10, 15 or 20 breeding cycles, selecting 100 parents with the highest GEBVs and crossing them at random. In the subsequent breeding cycles, the parents can be selected according to the deep scoping method or the HUC method with bridging. Additionally, both methods will also be able to select parents from a gene bank. Each simulation consists of 50 breeding cycles, and all results are averaged over 100 simulation runs.

### Truncation selection

Truncation selection selects 100 individuals with the highest GEBVs and couples them randomly. Breeders have been using truncation selection for centuries in the hope to pass favorable properties to the next generation. Unfortunately, this method also causes a strong reduction in the genetic variation. Therefore, truncation selection is an ideal and realistic method to simulate the loss of genetic variation in a breeding population as a result of selection.

### Haploid estimated breeding values

**Fig. 3:**
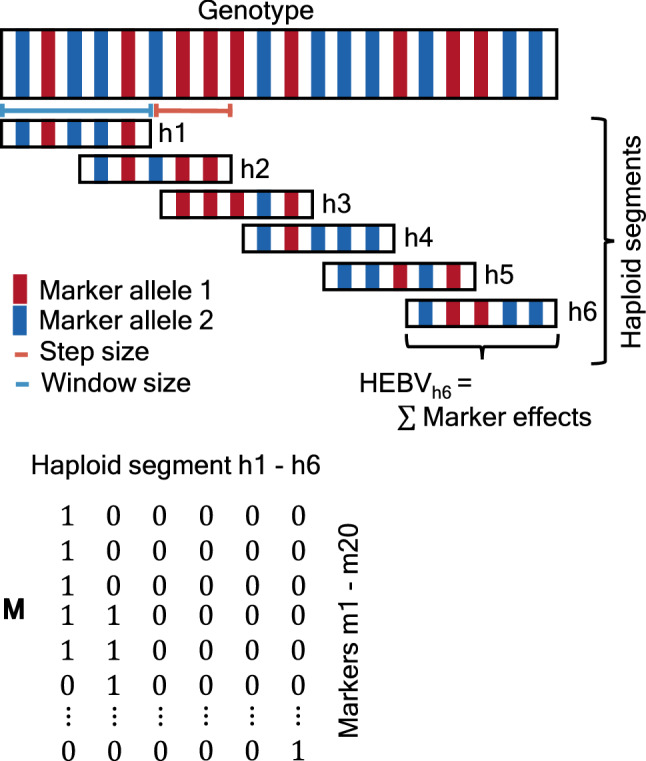
Haplotype is split into different haplotype segments containing an equal number of markers. Next, the HEBV is calculated per segment by summing up the marker effects of each segment separately

In plant breeding, GEBVs are commonly used to select the parental population. Daetwyler et al. (2015) proposed the OHV as an alternative selection metric in which the highest genetic value of each haplotype segment is used instead of the marker effects. In theory, a haplotype segment contains several alleles and markers that are always inherited together, but in the OHV approach, each chromosome is divided into different haplotype segments containing an equal number of markers. A diploid individual contains $n_H$ different haplotype segments and will have two haplotype values per segment representing the sum of the additive marker effects that are present in that segment on each homologous chromosome. The OHV is obtained by taking the sum of the highest haploid values per segment. In contrast to the GEBV, the OHV is better able to capture the potential benefits of heterozygous states in the breeding population. The HEBV proposed by Allier et al. (2020a) is similar to the OHV but allows for an overlap between the different haplotype segments. In this simulation study, the genotype is split into different haplotype segments containing 20 markers (window size) with an overlap of five markers (step size) (see Fig. 3). The same simulation parameters were adopted as reported by Allier et al. (2020a) and remained unchanged during the whole simulation study to allow for a fair comparison between the different methods. A matrix ${\mathbf {M}}$ of size $k\times n_H$, with $k$ the number of markers, is constructed to keep track of the selected markers per haplotype segment, such that $M_{ij}=1$ if marker $i$ is part of the $j$-th haplotype segment and $M_{ij}=0$ otherwise. Mathematically, the HEBV matrix ${{\mathbf {H}}}$ can be written as:

1 $$\begin{aligned} {{\mathbf {H}}}=({\mathbf {X}} \circ {\mathbf {1}}_{2n}\varvec{\beta }^{T}){\mathbf {M}}\, \end{aligned}$$

with ${\mathbf {X}}$ a matrix of size $2n\times k$ containing the haplotype of $n$ different individuals and $k$ different markers coded as 0 and 1 (such that the haplotype of individual *i* is represented at rows $2i-1$ and 2$i$), $\circ$ the Hadamard product operator, ${\mathbf {1}}_{2n}$ a vector of size 2$n$ containing 1s and $\varvec{\beta }$ a vector of size $k$ with estimated marker effects. Similar to the OHV, the HEBV between two individuals $i$ and $j$ is calculated as:

2 $$\begin{aligned} \mathrm {HEBV}(i,j)=\lambda \sum _{h=1}^{n_H} \max \left( {{\mathbf {H}}}_{2i-1,h}, {{\mathbf {H}}}_{2i,h}, {{\mathbf {H}}}_{2j-1,h}, {{\mathbf {H}}}_{2j, h} \right) \, \end{aligned}$$

with $\lambda$ a scaling parameter defined as the ratio between the step size and the window size. If the step size and window size are equal, then $\lambda = 1$ and the HEBV reduces to the OHV.

In a breeding population, an elite subpopulation (denoted *E*), containing individuals with high GEBVs, can be distinguished. The H-score $H(i)$ of an individual $i$ represents the maximal HEBV between this individual and any member of the elite subpopulation *E* (Allier et al. 2020a):

3 $$\begin{aligned} H(i) = \max _{j\in E} \mathrm {HEBV}(i,j)\,. \end{aligned}$$

In other words, an individual with a high H-score contains different favorable haplotype segments that are not available in the elite subpopulation (*E*) and should thus be selected as a parent.

### Deep scoping method

**Fig. 4:**
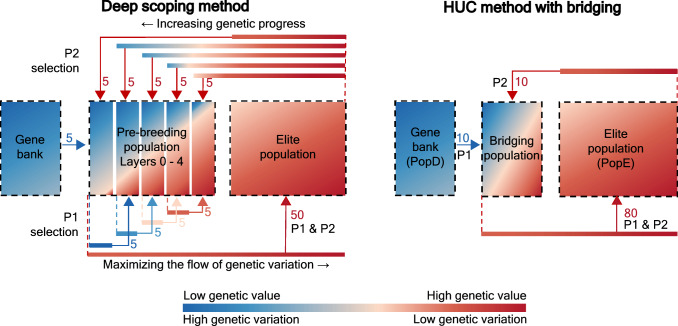
Left panel: overview of the deep scoping method. First, the individuals with the highest GEBVs are selected as elite parents. Next, for each layer, individuals from the elite population and the previous layer are selected. For Layer 0, elite individuals are combined with individuals of the gene bank. For each of the subsequent layers, the individuals can mature in the breeding population, increasing their genetic value, while the genetic variation is gradually decreased. Right panel: overview of the HUC method with bridging. First, the individuals with the highest GEBVs are selected as elite parents. Next, individuals of the gene bank with the highest H-scores are selected and crossed with individuals of the elite population containing the highest GEBVs

The deep scoping method combines truncation selection with the (re)introduction of (new) QTL alleles in the breeding population with the aim of maximizing both the short- and long-term genetic gain. To introduce new QTL alleles, a gene bank is used, containing a population with a high genetic variation, but lower mean genetic value. When individuals of the gene bank are introduced into the breeding population, their lower genetic value prevents them from being selected during truncation selection. This will create a gap between the genetic value of the elite individuals and the rest of the breeding population, isolating them from one another. Although both QTL alleles will still be present in the breeding population, the QTL alleles of the individuals in the elite population will still be fixed causing a premature convergence of the genetic value. Therefore, a three-step selection procedure was designed to not only introduce QTL alleles into the breeding population but also in the elite population. To do so, the breeding population is divided into two subpopulations: the elite population and the pre-breeding population (see Fig. 4). Individuals of the elite population are selected based on the highest GEBVs and are crossed to maximize short-term genetic gain. The selection of the pre-breeding population is divided into two steps: the selection for Layer 0 and the selection for Layers 1–4. For Layer 0, elite individuals are crossed with individuals from the gene bank to maximally introduce QTL alleles into the breeding population. The parental selection for the subsequent layers maximizes the flow of individuals between the pre-breeding population and the elite population, exploiting the genetic variation such that (new) favorable QTL alleles can be introduced into the elite population. Loosely inspired by deep learning (Ivakhnenko 1971), the deep scoping method uses different layers in which individuals flow from one layer to the next, in the hope that the information that was once present in the first layer can be useful in the future and thus be transferred to the elite population.

The breeding population consists of an elite (sub)population containing 500 individuals and a pre-breeding (sub)population containing five different layers with each 100 individuals. In order to create the elite population, 50 individuals with the highest GEBVs are selected. In contrast to truncation selection, the parents are not crossed at random. The individual with the highest GEBV is selected as the P1 parent and is coupled with a P2 parent that minimizes the genetic relationship between both parents. Other crossing block designs have been considered as well, such as crossing the two individuals with the highest GEBVs with each other or crossing the top-50 individuals with the top 51–100 individuals, but both designs resulted in a significantly lower long-term genetic gain.

The pre-breeding population tries to introduce favorable marker alleles into the breeding population and ultimately in the elite population. To select the first parents for Layer 0, the HEBVs for the individuals of the gene bank are calculated. Next, the H-score is calculated for each individual of the gene bank. The five individuals with the highest H-score and thus containing the most favorable haplotype segments are selected as P1 parents. The five P2 parents are selected from the elite population to maximize the genetic value of the offspring. To maximize the genetic variation of the offspring, the scoping method is used instead of truncation selection. The scoping method has been proposed by the present authors (Vanavermaete et al. 2020) and consists of two important steps: the pre-selection and the parental selection. The pre-selection will select a fraction of the breeding population containing individuals with the highest GEBVs. Next, each selected P1 parent is crossed with a pre-selected individual that maximizes the S-score between both parents. The S-score between two individuals $i$ and $j$ is computed as:

4 $$\begin{aligned} S(i,j)=\sum _{m=1}^{k}\text{ var }\{Z_{im},Z_{jm}\}p_{m}\,, \end{aligned}$$

with $k$ the number of markers, ${\mathbf {Z}}$ a matrix of size $n\times k$ containing the genotype of *n* selected individuals and *k* different markers coded as −1, 0, or 1 and ${\mathbf {p}}$ a vector of size $k$ with $p_{m}=0$ if both alleles of marker $m$ have been selected in the parental population or $p_{m}=1$ otherwise (Vanavermaete et al. 2020). An individual with a high S-score contains different marker alleles that are not yet present in the parental population and should thus be selected as a parent. It is possible that an individual of Layer 0 is selected as an elite P2 parent as long as it maximizes the genetic variation of the offspring.

The subsequent layers of the pre-breeding population gradually increase the genetic value of the Layer 0 individuals, while the genetic variation is slowly decreased such that favorable QTL alleles can be passed to the elite population. To allow for a continuous flow of favorable QTL alleles into the elite population, four additional layers are used. The effect of using a different number of layers will be discussed later (see Sect. “Flow from the pre-breeding population into the elite population”). In the subsequent layers, the P1 parents are selected from the previous layer, selecting individuals with the highest H-score. This ensures that individuals with favorable haplotype segments can flow to the next layer. The P2 parents are selected such that the genetic value of the offspring is maximized while preserving the genetic variation as much as possible. Individuals of previous layers are not considered as potential parents because they could reduce the genetic value of the offspring and thus interrupt the flow of QTL alleles in the breeding population. Both pre-selection and the S-score are used to select the P2 parent. First, based on the GEBV, candidate parents are pre-selected. Next, P2 parents are selected such that the S-score is maximized between both parents. In the parental selection for Layer 1, the top-400 individuals are pre-selected and can thus be used to select the P2 parents. In the parental selection for the subsequent layers, the number of individuals that are pre-selected decreases over each layer to increase the genetic gain. The parental selection for Layer 2 only pre-selects 300 individuals, followed by 200 and 100 individuals for the selection for Layer 3 and Layer 4, respectively. Again, it is possible that an elite parent is also selected as a pre-breeding parent as long as it maximizes the genetic variation of the offspring. The use of the scoping method during the parental selection helps to preserve the genetic variation, allowing for a slower but more accurate fixation of the QTL alleles. Individuals of the fourth and last layer should have the highest genetic values and could therefore be selected during truncation selection, finally introducing favorable QTL alleles into the elite population. Note that the elite population selects the individuals with the highest GEBVs over the entire breeding population, making it possible to select individuals of any layer into the elite population as long as the GEBV is high enough.

The implementation of the deep scoping method will require several breeding cycles. Starting with a truncation-selected breeding population, when the deep scoping method is used for the first time, the parental selection for Layer 0 crosses individuals of the elite population with individuals of the gene bank, but the parents of the subsequent layers will still be selected from truncation-selected individuals. In the next breeding cycle, the parental selection for Layer 1 crosses individuals of Layer 0 with individuals of the elite population, but the parents for Layers 2–4 will be selected from the offspring of truncation-selected individuals. For each layer that is used in the deep scoping method, one additional breeding cycle will be required before the deep scoping method becomes fully operational.

### HUC method with bridging

The HUC method combines the HEBV and the UC to select the parental population. A full description of the HUC method has been reported by Allier et al. (2020a). The HUC method combines an elite population (PopE) with a second donor population (PopD). The donor population is selected from a gene bank containing 500 different individuals. First, individuals with the highest GEBVs are selected as elite parents. Next, the individuals in the donor population with the highest H-scores are selected as donor parents. Next, a crossing block between the selected parents from the elite population and the donor population is built by maximizing the UC, which is calculated as:

5 $$\begin{aligned} U={\hat{\mu }}_{p}+i\rho {\hat{\sigma }}_{p}\,, \end{aligned}$$

with *U* the UC, ${\hat{\mu }}_{p}$ the predicted mean genetic value of the progeny, $i$ the selection intensity, $\rho$ the model performance and ${\hat{\sigma }}_{p}$ the predicted genetic variance of the progeny. Both parameters $i$ and $\rho$ are kept constant during the entire simulation. The UC was calculated using the implementation and parameter settings as published by Allier et al. (2020a) with $i = 2.06$ representing a selection intensity of 5% and $\rho =1$.

In our simulation study, the genetic value of the individuals of the gene bank is low. In such case, to allow for a fair comparison between the HUC method and the deep scoping method, the HUC method should be extended with a bridging population to assist the introduction of the individuals of the gene bank into the elite population (Allier et al. 2020b). This means that the breeding population is split into two parts: an elite population and a pre-breeding population. According to Allier et al. (2020b), 75% of the parental population is used to select the elite population, while the remaining 25% is used to select the pre-breeding individuals. Because the recurrent breeding scheme used in our simulation study requires the selection of an even number of parents, 80% of the parental population is used to select the elite population and the remaining 20% is used to select the pre-breeding population. In the elite population, 80 individuals with the highest GEBVs are selected and crossed using truncation selection as described in the deep scoping method. In the pre-breeding population, 10 elite individuals are crossed with 10 individuals of the gene bank (donors) according to the HUC method.

### Prediction model

The GEBVs are predicted by fitting a linear mixed effects model:

6 $$\begin{aligned} {\mathbf {y}}={\mathbf {1}}_{n} \beta + {\mathbf {Z}} {\mathbf {u}} + \varvec{\epsilon }\,, \end{aligned}$$

with ${\mathbf {y}}$ a vector of phenotypic values, ${\mathbf {1}}_{n}$ a vector of size $n$ containing 1s, $n$ the number of individuals in the training panel, $\beta$ the fixed effect (phenotypic mean), ${\mathbf {Z}}$ the incidence matrix of the training panel with marker information, ${\mathbf {u}}$ the marker effects following a normal distribution ${\mathcal {N}}({\mathbf {0}},{\mathbf {G}})$ with ${\mathbf {G}}=\sigma _{u}^{2}{\mathbf {I}}_{k}$ (with ${\mathbf {I}}_{k}$ the identity matrix of dimension $k$), $n$ the number of markers and $\varvec{\epsilon }$ the residual effects following a normal distribution ${\mathcal {N}}({\mathbf {0}},{\mathbf {R}})$ with ${\mathbf {R}}=\sigma _{e}^{2}{\mathbf {I}}_{n}$. Both variance components $\sigma _{u}^{2}$ and $\sigma _{e}^{2}$ are estimated by means of restricted maximum likelihood using the rrBLUP package (Endelman 2011). The GEBVs of the individuals are calculated as:

7 $$\begin{aligned} \hat{{\mathbf {g}}} = {\mathbf {Z}}\hat{{\mathbf {u}}}\,, \end{aligned}$$

with $\hat{{\mathbf {g}}}$ the GEBVs, ${\mathbf {Z}}$ the marker information and $\hat{{\mathbf {u}}}$ the predicted marker effects.

In breeding cycle 1, the complete base population is used as a training panel. In the subsequent breeding cycles, 150 individuals are phenotyped and added to the training panel according to the tails method, selecting 75 individuals based on the tails of the normally distributed GEBVs (Neyhart et al. 2017). According to Neyhart et al. (2017), the tails method delivers a nonsignificant higher genetic gain compared to other update methods. In the case of the deep scoping method, the tails method builds a training panel with elite individuals and pre-breeding individuals improving the prediction of GEBVs of the whole breeding population without the need for two separate prediction models. Each time the training panel is updated, 150 individuals that have been longest in the training panel are removed from the training panel to reduce computational time without reducing the prediction accuracy (Neyhart et al. 2017). To calculate the UC, the Markov chain Monte Carlo (MCMC) samples of the marker effects are required. This matrix is obtained by estimating the GEBVs that are used in the HUC method via the BGLR package using a Gibbs sampler with Gaussian prior (BRR) (Allier et al. 2020a; Pérez and de los Campos 2014).

### Simulation of the population

The simulation is built upon the work of Neyhart et al. (2017), using the packages GSSimTPUpdate and hypred in R (version 3.6.3). The dataset contains 1590 biallelic SNP markers from which 100 are selected as QTLs ($L=100$) and 1490 are used as markers to predict the genetic value. The true phenotypic value of the $i$-th individual ($y_{i}$) is calculated over three different environments:

8 $$\begin{aligned} y_{i}=\frac{1}{3}\sum _{j=1}^{3} g_{i}+e_{j}+\epsilon _{ij}\,, \end{aligned}$$

with $g_{i}$ the genetic value of the $i$-th individual, $e_j$ the $j$-th environmental effect, and $\epsilon _{ij}$ the residual effect of the $i$-th individual and the $j$-th environment. The genetic value is calculated by taking the sum of the QTL effects. The QTL effects are sampled from a geometric series such that at the $k$-th QTL, the favorable homozygote has a value of $a^k$, the unfavorable homozygote has a value of $-a^k$ and the heterozygote has a value of zero with $a=(L-1)/(L+1)$. Both the environmental and residual effects are drawn from a normal distribution with mean 0 and a variance component $\sigma _{E}^2$ and $\sigma _{e}^{2}$, respectively. The variance component of the environmental effect is defined as eight times the genetic variance, while the variance component of the residual effect is scaled to simulate a heritability of 0.5 (Bernardo 2014).

The simulation of the different breeding cycles is described by Vanavermaete et al. (2020). In this paper, a gene bank is added to the simulation. The gene bank is created by crossing individuals of the UMN population with individuals of the NDSU population. First, individuals of the UMN dataset are selected at random. For each parent, an individual of the NDSU dataset is selected that maximizes the S-score between both parents. The size of the gene bank is set at 500 individuals delivering a good balance between the preservation of the genetic variation of the base population and keeping the simulation time low.

### Data availability

The scripts, figures, datasets of the base population and supplementary data are available from the GitHub repository https://github.com/biointec/deep-scoping. The dataset and the simulation of the recurrent breeding cycle have been reported and published by Neyhart et al. (2017). The HUC method has been reported and published by Allier et al. (2020a).

## Results

### Truncation selection

**Fig. 5:**
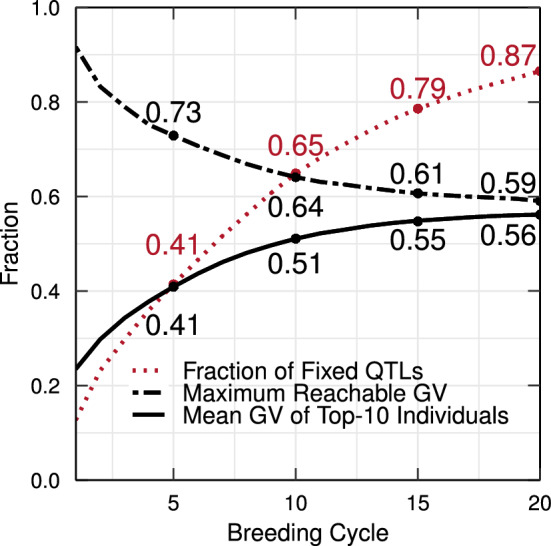
Simulation results of truncation selection over 20 breeding cycles. When truncation selection is used, the mean genetic gain of the top-10 individuals increases rapidly. Unfortunately, truncation selection also causes fixation of unfavorable QTL alleles, leading to a decrease in maximum reachable genetic value and causing a premature convergence of the mean genetic value of the top-10 individuals

To simulate a realistic initial breeding population, truncation selection is used to reduce the genetic variation. At breeding cycle zero, both alleles are present in the breeding population at 92% of the marker sites (see Fig. 5). Using truncation selection, the average genetic value of the breeding population increases while QTL alleles get fixed. The maximum reachable genetic value represents the sum of the QTL effects that are fixed (both favorable and unfavorable) and the sum of the favorable QTL effects that are not yet fixed. In other words, it represents the maximum genetic value that could still be reached, taking into account the fixation of unfavorable QTL alleles that has already occurred. A decrease in the maximum reachable genetic value, as observed in Fig. 5, indicates that favorable QTL alleles are eliminated from the breeding population. This causes a convergence to sub-optimal genetic values.

To assess different selection strategies, we consider the mean genetic value of only the top-10 individuals in the breeding population. This reflects the genetic value of the elite individuals that are candidates for commercialization. It allows for a better comparison between the different methods because the mean genetic value of the entire breeding population will be negatively influenced when individuals of a gene bank are introduced even if the genetic value of the elite individuals remains unchanged.

### Deep scoping method

**Fig. 6:**
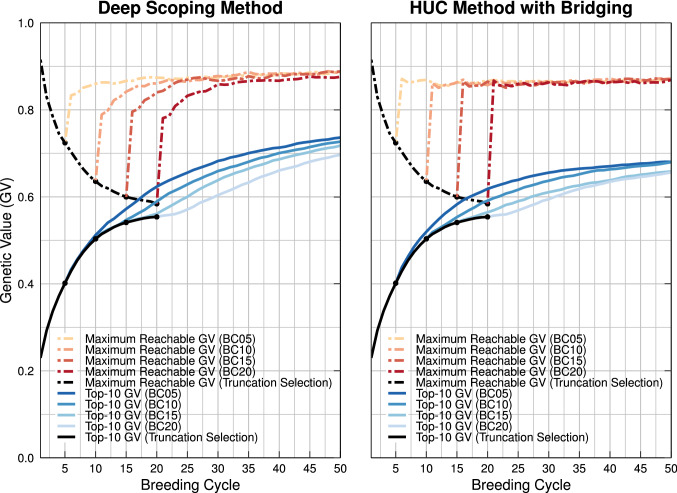
Simulation results of the deep scoping method and the HUC method with bridging starting at breeding cycles 5, 10, 15, and 20. Prior to the selection methods, truncation selection is used to reduce the genetic variation of the breeding population (black line). By deploying a gene bank, new QTL alleles are introduced into the breeding population, increasing the maximum reachable genetic value and avoiding a premature convergence of the genetic value. Compared to the HUC method with bridging, the deep scoping method can introduce more QTL alleles into the breeding population leading to a higher maximum reachable genetic value. The deep scoping method reaches a higher mean genetic values of the top-10 individuals in the long term compared to the HUC method with bridging

The deep scoping method relies on a gene bank to introduce new genetic material into the breeding population. Next, the genetic value of the pre-breeding individuals is increased facilitating their transition into the elite population. In the first scenario, the deep scoping method is used after five breeding cycles of truncation selection (Population BC05). Once the gene bank is available, the newly introduced marker alleles lead to an increase in the maximum reachable genetic value (see Fig. 6). In the short term, the deep scoping method reaches the same genetic values as truncation selection. However, truncation selection results in a premature convergence, while the genetic value of the deep scoping method continues to increase, resulting in an 18% points higher genetic value in the long term (see Tables S1 and S2). In the other scenarios, truncation selection is used for 10, 15, or even 20 breeding cycles, resulting in a breeding population with a higher number of fixed QTL alleles. Again, when the deep scoping method is used, the maximum reachable genetic value increases rapidly. The genetic value differs according to the starting point at which the deep scoping method is first invoked. The longer truncation selection is used, the longer it takes before a breeding population reaches a certain genetic value. Certainly, when 20 breeding cycles of truncation selection have been used, several breeding cycles of the deep scoping method are needed before the genetic value can escape from the local optimum. In the (very) long term, the four different scenarios will converge to the same value.

**Fig. 7:**
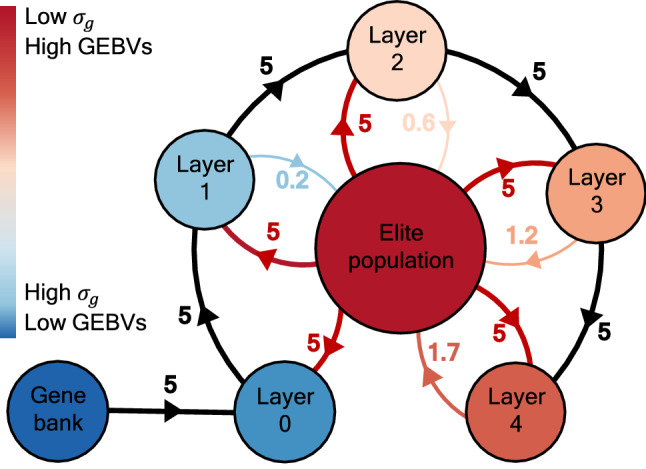
Overview of how individuals flow between the different subpopulations when the deep scoping method is applied on a breeding population after five initial breeding cycles of truncation selection. A color scheme is used to indicate the change in genetic value and genetic variation of the different subpopulations. The black and dark red arrows represent the selection of respectively the first and second parent. From the first two layers, very few individuals are selected into the elite population. After progressing over three to four layers, an average of one to two parents per breeding cycle is accepted into the elite population

In Fig. 7, the flow of individuals between the different layers of the deep scoping method after five initial breeding cycles of truncation selection is illustrated. The genetic value of the individuals over the first two layers is still too low, limiting their selection into the elite population. The individuals of Layers 3–4 have a higher genetic value, allowing for the transition of approximately one to two individuals into the elite population over each breeding cycle.

### HUC method with bridging

The HUC method with bridging combines two different parental selection schemes. On the one hand, elite individuals with the highest GEBVs are selected and crossed with each other, maximizing the short-term genetic gain. On the other hand, individuals of the gene bank are crossed with elite individuals to introduce QTL alleles into the breeding population. By crossing the individuals of the gene bank with an elite individual, the GEBVs of the offspring increase such that they can be selected as an elite parent in the next breeding cycle, maximizing the long-term genetic gain. However, when the GEBVs of the breeding population increase, the gap between the genetic value of the elite individuals and the gene bank increases as well, impeding the transition of pre-breeding individuals into the elite population and thus resulting in the convergence to a sub-optimal genetic value. Increasing the number of initial breeding cycles using truncation selection (e.g., Population BC20) increases the gap between the genetic value of the breeding population and the gene bank, resulting in the convergence to even lower genetic values (see Fig. 6). The mean genetic values of the top-10 individuals of the proposed methods are reported in Tables S1 and S2.

### Robustness of the deep scoping method

**Fig. 8:**
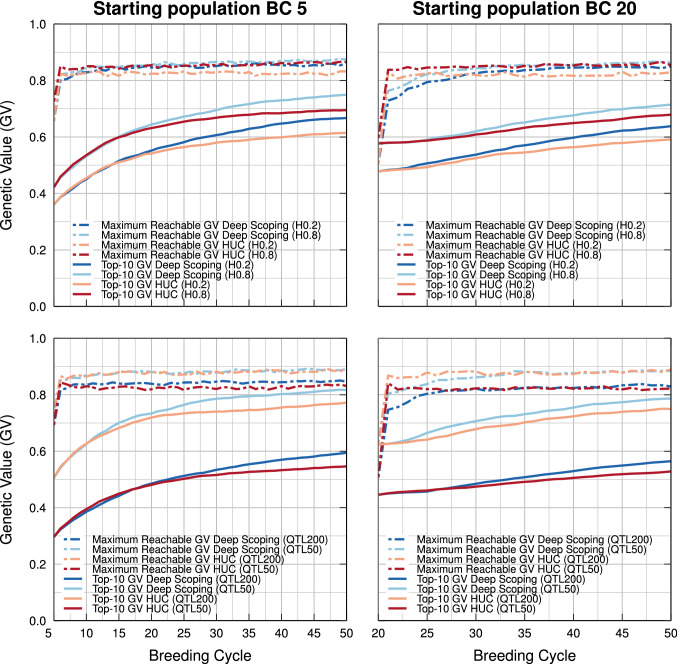
Simulation results of the deep scoping method and the HUC method with bridging for a heritability of 0.2 and 0.8 using 100 QTLs (top) and for a heritability of 0.5 using 50 and 200 QTLs (bottom line). The impact of both methods on the genetic value and on the maximum reachable genetic value is shown after 5 (left) and 20 (right) breeding cycles of truncation selection

The deep scoping method has been tested in different simulation settings. Each experiment consists of testing 100 different genomes such that the effects of using the deep scoping method or the HUC method with bridging can be studied using different QTL and marker positions. The effect of the heritability and of the number of QTLs on the genetic gain of both methods have also been tested and are shown in Fig. 8. Simulation studies were performed using a heritability of 0.2, 0.5, and 0.8 using 100 different QTLs, and a heritability of 0.5 using 50, 100, and 200 QTLs. In all six cases, the deep scoping method resulted in higher genetic values in the long term compared with the HUC method with bridging. Regardless of the heritability, number of QTLs, or the QTL and marker positions, the deep scoping method outperformed the HUC method with bridging in the long term.

## Discussion

### Introducing and preserving the genetic variation in the breeding population

The primary goal of the deep scoping method is to preserve newly introduced marker alleles in the breeding population by allowing for a gradual flow of genetic material from the pre-breeding population into the elite population. To achieve this, the scoping method has been redesigned. In the original scoping method, the first parent was greedily selected to prioritize the genetic progress, while the second parent was selected by maximizing the S-score between both parents, preserving the genetic variation of the breeding population. In the deep scoping method, the individuals with the highest H-scores are selected as P1 parents, prioritizing the preservation of favorable marker alleles in the breeding population. Similar to the scoping method, the second parent is selected by maximizing the S-score between both parents. Since both the H-score and the S-score quantify genetic variation, the second parent is taken from a pre-selected population that contains individuals with the highest GEBVs. This way, genetic progress is maximized as well, facilitating the transition of pre-breeding individuals into the elite population. Moreover, by using the S-score, both alleles of each marker will be preserved in the breeding population to the extent possible, thus minimizing the loss of (favorable) QTL alleles (Vanavermaete et al. 2020). By decreasing the size of the pre-selection fraction over each layer, the genetic variation will gradually decrease over each layer while the average genetic value increases.

The deep scoping method continuously introduces genetic material into the breeding population and therefore, the loss of genetic variation of the elite population is not an issue. In the case of the scoping method, when an unfavorable QTL allele was fixed in the breeding population, the maximum reachable genetic value was reduced causing a lower genetic value in the long term (Vanavermaete et al. 2020). In the case of the deep scoping method, the different QTL alleles are still preserved in the pre-breeding population or the gene bank, and when an unfavorable QTL allele is fixed in the elite population, it is thus still possible to introduce the corresponding favorable QTL allele into the next breeding cycles. Nevertheless, enforcing the preservation of genetic variation remains important to maximize the genetic gain over each breeding cycle.

### Population size and required resources

The deep scoping method does not require a separate pre-breeding program and is able to improve the individuals of the gene bank with the same resources as a breeding program without pre-breeding, minimizing the cost of the deep scoping method. This means that with the same population size (and resources) as truncation selection, the deep scoping method reintroduces genetic variation into the breeding population and maximizes the genetic gain thereof. Only the gene bank could be seen as an additional investment. The gene bank contains 500 individuals that are created by crossing individuals of both the UMN and the NDSU dataset by maximizing the genetic variation between both crosses. This means that the individuals of the gene bank will have different heterozygous markers and low genetic values. This way, the ability to include low-GEBV individuals of both the deep scoping method and the HUC method with bridging was studied. Including more exotic germplasm could require more layers, whereas the inclusion of individuals with a higher genetic value could also be done with a lower number of layers.

### GEBVs versus HEBVs

In genomic selection, GEBVs are often used to select the parental population. To do so, a mixed effect model is used to predict the marker effects, which are used to calculate the GEBVs according to Eq. (7). The genotype of an individual is often represented by bi-allelic markers coded as -1, 0, or 1. Assuming a positive marker effect $u_m$, the $m$-th marker of an homozygous individual $i$ will yield a positive contribution if the individual carries the reference allele ($Z_{im}=1$) or a negative contribution if the alternative allele ($Z_{im}=-1$) is present. If the individual is heterozygous ($Z_{im}=0$), meaning that both alleles are present once, the marker does not affect the GEBV despite the fact that it still has a 50% probability to pass the favorable marker allele to the next generation. In other words, the GEBVs penalize heterozygous markers despite the fact that they contain favorable marker alleles. Taking into account that the genetic information of thousands of markers is reduced to a single value, unfavorable QTL alleles could be fixed into the breeding population when their negative marker effect is masked by many other positive QTL effects, or favorable QTL alleles could be eliminated from the breeding population when their QTL effects are masked by many other negative QTL effects.

A parental selection based on GEBVs could also lead to the selection of closely related individuals, fixating several favorable and unfavorable QTL alleles in the breeding population. This could be avoided by penalizing the GEBVs to minimize the rate of inbreeding and reducing the loss in genetic variation (Cervantes et al. 2016; Akdemir and Sánchez 2016). Nevertheless, reducing the genetic information of thousands of markers into a single value remains a major disadvantage of the GEBVs and therefore, the HEBVs are used instead. In contrast to GEBVs, HEBVs split the genotype into different haplotypes and each haplotype is compared among the selected individuals. The available genotypic information is no longer reduced into a single value, lowering the probability to mask certain QTL effects and thus avoiding the elimination of one or more favorable QTL alleles. The HEBV score is calculated by taking the maximum genetic value between the haplotype segments of different individuals, which means that heterozygous alleles could have the same contribution as the favorable homozygous marker, preserving all the favorable QTL alleles in the breeding population. The H-score is an extension of the HEBV and scores the ability of an individual to bring new favorable haplotype segments into the elite population, maximizing the genetic preservation by both the HUC method with bridging and the deep scoping method.

### Comparison of the HUC method and the deep scoping method

As both the HUC method and the deep scoping method rely on the use of a gene bank, it is possible to make a fair comparison between these methods. The HUC method with bridging is a combination of, on the one hand, the HUC method and, on the other hand, a breeding scheme with a bridging population (Allier et al. 2020a, b). Originally, the HUC method selects individuals based on the H-score in order to cross elite individuals with a donor population. The donor population contains all the individuals of the breeding population that are not selected in the elite population and the individuals of the gene bank. The elite population often contains closely related individuals with low genetic variation; therefore, individuals of the gene bank will have a high H-score and will be selected as a parent, reducing the genetic value of the offspring. Originally, the HUC method was designed to introduce individuals with a similar or slightly lower genetic value than the elite population. However, when a gene bank is used containing individuals with low GEBVs, the HUC method fails to increase the genetic value of the breeding population. For this case, Allier et al. (2020b) designed a new breeding scheme that incorporates a bridging population. However, this breeding scheme uses the GEBVs to select the parental population. By using the HUC method in a breeding scheme with bridging, we were able to compare the deep scoping method with the HUC method, both using the HEBVs as selection criterion.

Both the HUC method with bridging and the deep scoping method use truncation selection to maximize the short-term genetic gain. The deep scoping method consists of two different populations: the elite population and the pre-breeding population, which is built up out of five different layers. The size of the elite population will be smaller compared to that of the HUC method with bridging, which only contains two populations: the elite population and the bridging population. In the HUC method with bridging, ten individuals of the gene bank and ten individuals of the elite population are crossed. The deep scoping method only selects five individuals of the gene bank and crosses them with the elite individuals. Therefore, the HUC method with bridging will be able to reintroduce more genetic variation into the breeding population after one breeding cycle. The deep scoping method will need several breeding cycles before reaching the same maximum reachable genetic value as the HUC method with bridging. As long as pre-breeding individuals are not selected in the elite population, the same individuals of the gene bank will be selected into Layer 0, therefore, two breeding cycles of deep scoping will not result in the same amount of genetic variation as observed after one breeding cycle using the HUC method with bridging. This also explains why, when the deep scoping method starts at breeding cycle five, a higher increase in the maximum reachable genetic value is observed for the HUC method with bridging. Increasing the size of Layer 0 in the deep scoping method may increase the maximum reachable genetic value in a similar way as in the HUC method with bridging, but because it does not influence the genetic gain in the short or the long term, increasing the size of Layer 0 is not necessary.

**Fig. 9:**
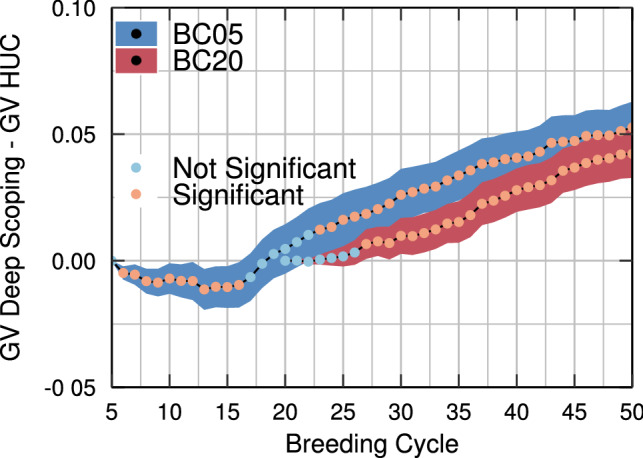
Mean genetic value of the top-10 individuals is compared between the deep scoping method and the HUC method with bridging using a paired sampled t-test, for two scenarios with respectively 5 and 20 initial breeding cycles of truncation selection. The difference in mean genetic value with a 95% confidence interval is reported. The color of each dot indicates whether the difference in genetic value between both methods is significant ($p<0.05$)

The pre-breeding is similar in both methods, however, the HUC method with bridging builds a crossing block by maximizing the UC, whereas the deep scoping method does this by using the S-score. In other words, the HUC method pairs up parents to maximize the genetic gain of the offspring, while the deep scoping method pairs up parents to maximize the genetic variation of the offspring. The deep scoping method also introduces four additional layers that are used to guide the development of pre-breeding individuals into elite individuals and thus to facilitate the flow of (favorable) QTL alleles into the elite population. In the HUC method with bridging, the selected individuals of the gene bank only have one breeding cycle to be selected as elite individuals. Therefore, when the difference in genetic value between the gene bank and the breeding population increases, the transition of pre-breeding individuals into the elite population degrades, causing a premature convergence of the genetic value.

Although the deep scoping method results in a 4–6% points higher long-term genetic gain compared with the HUC method with bridging, in the short term, a slightly lower genetic value is observed (see Fig. 9). The elite population of the HUC method represents 80% of the breeding population, whereas in the deep scoping method, only 50% of the breeding population is used. Therefore, when both methods are used after five breeding cycles of truncation selection, the HUC method will be able to select more elite individuals to convert the remaining genetic variation of the breeding population into genetic gain resulting in significantly higher genetic values in the short term compared with the deep scoping method. The HUC method with bridging also selects more individuals from the gene bank, increasing the genetic variation in the breeding population, which also contributes to the maximization of the short-term genetic gain. However, when both the HUC method with bridging and the deep scoping method are used after 20 breeding cycles of truncation selection, the genetic variation of the breeding population has been reduced and the HUC method with bridging will be unable to gain higher short-term genetic values compared with the deep scoping method.

### Flow from the pre-breeding population into the elite population

The deep scoping method uses different layers to guide the genetic progress of the pre-breeding individuals and to facilitate their transition into the elite population. Each layer selects parents from the previous layer and couples them with an individual of the elite population, maximizing the genetic gain of the offspring. Prior to the deep scoping method, truncation selection is used to simulate a realistic breeding population. When the deep scoping method is introduced in the breeding program, an additional breeding cycle per layer will be required before the deep scoping method becomes fully operational. Once that is done, the number of layers represents the number of breeding cycles a pre-breeding individual is allowed to be crossed with an elite individual to increase the genetic value of their offspring and thus to pass on their genotypic information into the elite population. Individuals that are not selected in Layer 4 will be eliminated from the breeding population.

**Fig. 10:**
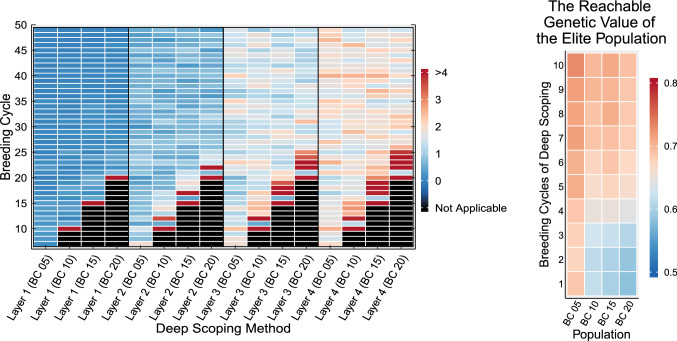
Left: an overview of the mean number of individuals that are selected in the elite population for each subpopulation. Right: the maximum reachable genetic value of the elite population for the first ten breeding cycles of deep scoping after 5, 10, 15 and 20 breeding cycles of truncation selection

Figure 10 illustrates the flow of pre-breeding individuals into the elite population. In the first layer, individuals are rarely accepted into the elite population, except for the first breeding cycle after the introduction of the deep scoping method. This indicates that the use of a single layer (like in the HUC method with bridging) is insufficient to properly introduce pre-breeding individuals into the elite population. In the second layer, the transition of individuals into the elite population is still limited and it is only after progressing over three or four layers that a more substantial flow of pre-breeding individuals into the elite population can be observed. Each layer that is added to the deep scoping method allows for a better development of the pre-breeding individuals, which will facilitate their transition into the elite population.

**Fig. 11:**
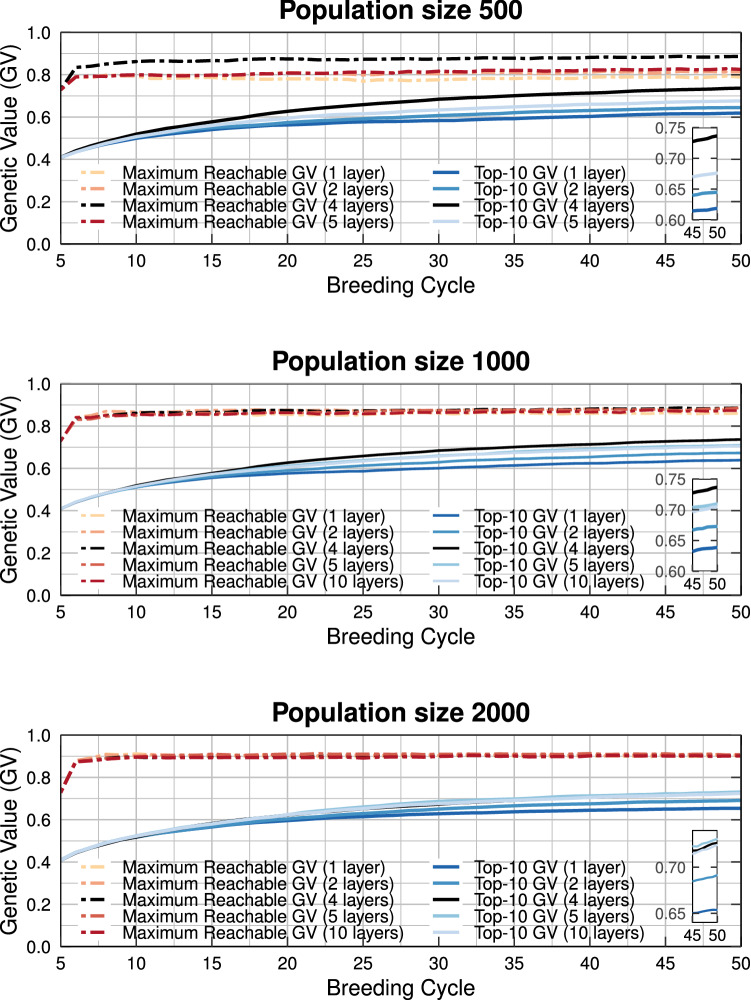
Simulation results of the deep scoping method using 1–10 different layers for a population size of 500, 1000, and 2000 individuals. Increasing the number of layers often results in higher long-term genetic gains. However, if the number of individuals per layer is too low, the long-term genetic gain is reduced

In Fig. 11, the genetic value is shown for a population size of 500, 1000, and 2000 individuals using a different number of layers. Increasing the number of layers in a breeding population will decrease the number of individuals (and parents) per layer, but will allow for more time to increase the genetic value of the pre-breeding individuals increasing the flow of individuals from the gene bank to the elite population. Therefore, increasing the number of layers will often result in higher long-term genetic gains. Nevertheless, when the number of individuals per layer becomes too small, the probability to develop potentially interesting individuals is reduced, causing a lower long-term genetic gain. This can be avoided by increasing the size of the parental population, but that will also increase the total financial cost.

### Implementing the deep scoping method

**Fig. 12:**
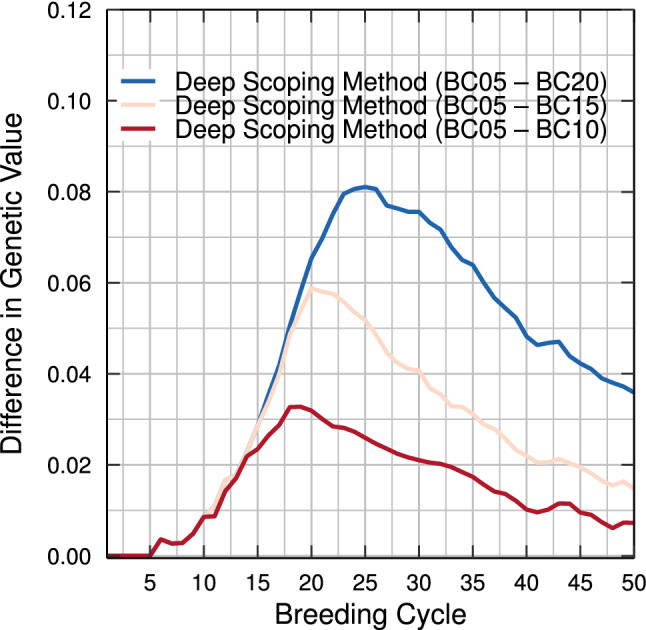
Difference in genetic value between the deep scoping method using two different breeding populations. The sooner truncation selection is replaced by the deep scoping method, the sooner the genetic gain of the top-10 individuals will increase until the breeding value converges to the same value in the long term

Regardless of the fact whether the breeding population underwent 5, 10, 15, or 20 breeding cycles of truncation selection, when the deep scoping method is used, for each layer, a similar flow of individuals to the elite population is observed (see Fig. 10). The longer truncation selection is used prior to the deep scoping method, the longer it will take to reach the same long-term genetic gain (see Fig. 12). When the deep scoping method is initiated after only five breeding cycles of truncation selection, the different layers, still filled with the offspring of truncation-selected individuals, will be able to maximize the genetic value in the short term until the pre-breeding individuals are introduced to maximize the long-term genetic gain in the elite population. However, when the genetic variation of the breeding population is reduced after, e.g., 20 breeding cycles of truncation selection, the genetic value of the base population has already converged. At that point, at least five breeding cycles will be required before individuals of the pre-breeding population will be accepted in the elite population, reintroducing genetic variation in the elite population that will allow the breeding population to escape from the local optimum (see Fig. 10 right). If the deep scoping method would have been invoked after only five breeding cycles of truncation selection, the mean genetic value of the top-10 individuals would have been 8% points higher at breeding cycle 25 (see Fig. 12). In other words, the sooner the deep scoping method is adopted in a breeding program, the sooner the breeding population will produce higher genetic gains compared with the same breeding population using truncation selection.

Even compared with the scoping method, using the deep scoping method after five breeding cycles of truncation selection will result in a 3% points higher genetic value in the long term (see Table S1) (Vanavermaete et al. 2020). While the scoping method is able to reach high long-term genetic gains, the method is not perfect and allows for the fixation of a few unfavorable QTL alleles. Therefore, methods like deep scoping that can reintroduce genetic variation into the breeding population are important to maximize the genetic gain and avoid a premature convergence of the genetic value.

### The size of the gene bank

Both the deep scoping method and the HUC method with bridging use a gene bank to reintroduce genetic variation into the breeding population. Increasing the size of the gene bank had almost no effect on the mean genetic value of the top-10 individuals. Only when the size of the gene bank was reduced to 200 individuals, a reduction of the genetic value in the long term was observed. At that point, the gene bank was too small to contain both QTL alleles, reducing the introduction of genetic variation into the breeding population. In this simulation setting, a gene bank is essential to maximize the genetic gain in the long term, but it is not necessary to collect thousands of different individuals. As long as all the QTL alleles are present in the gene bank, the deep scoping method will be able to maximize the long-term genetic gain.

### Conclusion

Truncation selection often reduces the genetic variation of the breeding population in its striving for a maximal genetic value of the breeding population, to the extent that the adoption of variation-preserving methods such as the population merit and scoping methods is rather pointless. To increase the genetic variation of the breeding population, a gene bank containing a broad genetic variation is needed. However, when the gap between the genetic value of the breeding population and the gene bank increases, the transition of pre-breeding individuals into the elite population becomes difficult. The deep scoping method uses different layers of pre-breeding individuals to maximize the flow of pre-breeding individuals into the elite population, thereby introducing (favorable) QTL alleles into the elite population. Replacing the frequently used GEBVs by HEBVs allows for a more accurate selection of individuals containing favorable QTL alleles, maximizing the long-term genetic gain. In summary, the deep scoping method combines an elite population with different layers using the HEBVs, H-score, and S-score, thus maximizing the genetic gain in the short as well as in the long term without increasing the financial cost of the breeding program.

## Supplementary Information

Below is the link to the electronic supplementary material.

Supplementary file1 (PDF 68 kb)
