# Supplementary material for: Deep scoping: a breeding strategy to preserve, reintroduce and exploit genetic variation
Source: Theor Appl Genet. 2021 Aug 13;134(12):3845–61. doi: 10.1007/s00122-021-03932-w (PMC8580937; doi:10.1007/s00122-021-03932-w)
Supplement: Supplementary file 1 — Supplementary file1 (PDF 68 kb) [file 122_2021_3932_MOESM1_ESM.pdf]

Table S1 The mean genetic value and the standard deviation of the top-10 individuals for truncation selection, the scoping method and the population merit method over 250 experiments as published by Vanavermaete et al. (2020)

Breeding Cycle	Truncation selection	Scoping Method (SR = 0.3)	Population Merit Method (c = 20)
1	0.23 ± 0.08	0.23 ± 0.08	0.23 ± 0.08
3	0.34 ± 0.08	0.34 ± 0.07	0.34 ± 0.08
5	0.41 ± 0.09	0.41 ± 0.07	0.40 ± 0.08
7	0.46 ± 0.08	0.47 ± 0.07	0.45 ± 0.08
9	0.50 ± 0.08	0.51 ± 0.07	0.49 ± 0.07
11	0.52 ± 0.08	0.55 ± 0.07	0.52 ± 0.08
13	0.54 ± 0.08	0.57 ± 0.07	0.54 ± 0.07
15	0.55 ± 0.08	0.60 ± 0.07	0.56 ± 0.07
17	0.55 ± 0.08	0.62 ± 0.07	0.57 ± 0.07
19	0.56 ± 0.08	0.63 ± 0.07	0.58 ± 0.07
21	0.56 ± 0.08	0.65 ± 0.07	0.60 ± 0.07
23	0.56 ± 0.08	0.66 ± 0.07	0.60 ± 0.07
25	0.56 ± 0.08	0.66 ± 0.07	0.61 ± 0.07
27	0.56 ± 0.08	0.67 ± 0.07	0.61 ± 0.07
29	0.56 ± 0.08	0.68 ± 0.07	0.62 ± 0.07
31	0.56 ± 0.08	0.68 ± 0.07	0.62 ± 0.07
33	0.56 ± 0.08	0.69 ± 0.07	0.63 ± 0.07
35	0.56 ± 0.08	0.69 ± 0.07	0.63 ± 0.07
37	0.56 ± 0.08	0.70 ± 0.07	0.63 ± 0.07
39	0.56 ± 0.08	0.70 ± 0.07	0.63 ± 0.07
41	0.56 ± 0.08	0.70 ± 0.07	0.63 ± 0.07
43	0.56 ± 0.08	0.71 ± 0.07	0.63 ± 0.07
45	0.56 ± 0.08	0.71 ± 0.07	0.64 ± 0.07
47	0.56 ± 0.08	0.71 ± 0.07	0.64 ± 0.07
49	0.56 ± 0.08	0.71 ± 0.07	0.64 ± 0.07
50	0.56 ± 0.08	0.71 ± 0.07	0.64 ± 0.07

Table S2 The mean genetic value and the standard deviation of the top-10 individuals for the deep scoping method and the HUC method with bridging over 100 experiments.

BC	Deep scoping (BC05)	HUC (BC05)	Deep scoping (BC10)	HUC (BC10)	Deep scoping (BC15)	HUC (BC15)	Deep scoping (BC20)	HUC (BC20)
1	-	-	-	-	-	-	-	-
3	-	-	-	-	-	-	-	-
5	0.40 ± 0.09	0.40 ± 0.09	-	-	-	-	-	-
7	0.46 ± 0.09	0.46 ± 0.09	-	-	-	-	-	-
9	0.49 ± 0.08	0.50 ± 0.08	-	-	-	-	-	-
11	0.53 ± 0.08	0.53 ± 0.08	0.52 ± 0.09	0.52 ± 0.09	-	-	-	-
13	0.55 ± 0.08	0.56 ± 0.08	0.53 ± 0.09	0.54 ± 0.09	-	-	-	-
15	0.57 ± 0.08	0.58 ± 0.08	0.55 ± 0.08	0.55 ± 0.09	0.54 ± 0.09	0.54 ± 0.09	-	-
17	0.59 ± 0.08	0.60 ± 0.07	0.56 ± 0.08	0.57 ± 0.09	0.55 ± 0.09	0.55 ± 0.09	-	-
19	0.61 ± 0.08	0.61 ± 0.07	0.58 ± 0.08	0.59 ± 0.09	0.56 ± 0.09	0.56 ± 0.09	-	-
21	0.63 ± 0.08	0.62 ± 0.07	0.60 ± 0.08	0.60 ± 0.09	0.57 ± 0.09	0.57 ± 0.09	0.56 ± 0.09	0.56 ± 0.09
23	0.64 ± 0.08	0.63 ± 0.07	0.61 ± 0.08	0.61 ± 0.09	0.58 ± 0.08	0.58 ± 0.09	0.56 ± 0.09	0.56 ± 0.09
25	0.66 ± 0.08	0.64 ± 0.07	0.63 ± 0.08	0.62 ± 0.09	0.60 ± 0.08	0.59 ± 0.09	0.57 ± 0.09	0.57 ± 0.09
27	0.67 ± 0.08	0.65 ± 0.07	0.64 ± 0.08	0.63 ± 0.08	0.62 ± 0.08	0.60 ± 0.09	0.59 ± 0.08	0.58 ± 0.09
29	0.68 ± 0.07	0.65 ± 0.07	0.65 ± 0.08	0.63 ± 0.08	0.63 ± 0.08	0.61 ± 0.08	0.60 ± 0.08	0.59 ± 0.09
31	0.69 ± 0.07	0.66 ± 0.07	0.66 ± 0.08	0.64 ± 0.08	0.65 ± 0.08	0.61 ± 0.09	0.61 ± 0.08	0.60 ± 0.09
33	0.69 ± 0.07	0.66 ± 0.07	0.67 ± 0.08	0.65 ± 0.08	0.66 ± 0.08	0.62 ± 0.08	0.62 ± 0.08	0.61 ± 0.08
35	0.70 ± 0.07	0.66 ± 0.07	0.68 ± 0.07	0.65 ± 0.08	0.66 ± 0.08	0.63 ± 0.08	0.63 ± 0.08	0.62 ± 0.08
37	0.71 ± 0.07	0.67 ± 0.07	0.69 ± 0.08	0.66 ± 0.08	0.67 ± 0.08	0.63 ± 0.08	0.65 ± 0.08	0.62 ± 0.08
39	0.71 ± 0.07	0.67 ± 0.07	0.70 ± 0.08	0.66 ± 0.08	0.68 ± 0.08	0.64 ± 0.08	0.66 ± 0.08	0.63 ± 0.08
41	0.72 ± 0.07	0.67 ± 0.07	0.70 ± 0.08	0.66 ± 0.08	0.69 ± 0.08	0.64 ± 0.08	0.66 ± 0.08	0.64 ± 0.08
43	0.72 ± 0.07	0.67 ± 0.07	0.71 ± 0.07	0.67 ± 0.08	0.70 ± 0.08	0.65 ± 0.08	0.67 ± 0.08	0.64 ± 0.08
45	0.73 ± 0.07	0.68 ± 0.07	0.71 ± 0.07	0.67 ± 0.08	0.70 ± 0.08	0.65 ± 0.08	0.68 ± 0.08	0.65 ± 0.08
47	0.73 ± 0.07	0.68 ± 0.06	0.72 ± 0.07	0.67 ± 0.08	0.71 ± 0.08	0.65 ± 0.08	0.69 ± 0.08	0.65 ± 0.08
49	0.73 ± 0.06	0.68 ± 0.07	0.72 ± 0.07	0.68 ± 0.08	0.71 ± 0.08	0.66 ± 0.08	0.69 ± 0.08	0.65 ± 0.08
50	0.74 ± 0.07	0.68 ± 0.07	0.73 ± 0.07	0.68 ± 0.08	0.72 ± 0.08	0.66 ± 0.08	0.70 ± 0.08	0.66 ± 0.08

References

Vanavermaete D, Fostier J, Maenhout S, De Baets B (2020) Preservation of genetic variation in a breeding population for long-term genetic gain. G3 10(8):2753–2762
